# Association of pulse rate with outcomes in heart failure with reduced ejection fraction: a retrospective cohort study

**DOI:** 10.1186/s12872-020-01384-6

**Published:** 2020-02-26

**Authors:** Katherine E. Kurgansky, Petra Schubert, Rachel Parker, Luc Djousse, Jerome B. Riebman, David R. Gagnon, Jacob Joseph

**Affiliations:** 1 Massachusetts Veterans Epidemiology and Research Information Center (MAVERIC), Veterans Affairs Boston Healthcare System, Boston, MA USA; 2 Department of Medicine, Division of Aging, Brigham and Women’s Hospital, Harvard Medical School, Boston, MA USA; 3 Amgen Inc., Thousand Oaks, CA USA; 4 Department of Biostatistics, Boston University School of Public Health, Boston, MA USA; 5 Department of Medicine, Division of Cardiovascular Medicine, Brigham and Women’s Hospital, Harvard Medical School, Boston, MA USA; 6 Cardiology Section, VA Boston Healthcare System, 1400 VFW Parkway, West Roxbury, MA 02132 USA

**Keywords:** Heart failure, Heart rate, Outcomes, Beta-blocker, Hospitalization, Mortality

## Abstract

**Background:**

In a real-world setting, the effect of pulse rate measured at the time of diagnosis and serially during follow-up and management, on outcomes in heart failure with reduced ejection fraction (HFrEF), has not been well-studied. Furthermore, how beta-blockade use in a real-world situation modifies this relation between pulse rate and outcomes in HFrEF is not well-known. Hence, we identified a large, national, real-world cohort of HFrEF to examine the association of pulse rate and outcomes.

**Methods:**

Using Veterans Affairs (VA) national electronic health records we identified incident HFrEF cases between 2006 and 2012. We examined the associations of both baseline and serially measured pulse rates, with mortality and days hospitalized per year for heart failure and for any cause, using crude and multivariable Cox proportional hazards and Poisson or negative binomial models, respectively. The exposure was examined as continuous, dichotomous, and categorical. Post-hoc analyses addressed the interaction of pulse rate and beta-blocker target dose.

**Results:**

We identified 51,194 incident HFrEF cases (67 ± 12 years, 98% male, 77% white. A significant positive, near linear relationship was observed for both baseline and serially measured pulse rates with all-cause mortality, all-cause hospitalization and heart failure hospitalization after adjusting for covariates including beta-blocker use. Patients who had a pulse rate ≥ 70 bpm in the past 6 months had 36% (95% CI: 31–42%), 25% (95% CI: 19–32%), and 51% (95% CI: 33–72%) increased rates of mortality, all-cause hospitalization, and heart failure hospitalization, respectively, compared to patients with pulse rates < 70 bpm. A minority of subjects (15%) were treated with guideline directed beta blockade ≥50% of recommended target dose, among whom better outcomes were seen compared to those who did not achieve target dose in patients with pulse rates both above and below 70 beats per minute.

**Conclusions:**

High pulse rate, both at the time of diagnosis and during follow-up, is strongly associated with increased risk of adverse outcomes in HFrEF patients, independent of the use of beta-blockers. In a real-world setting, the majority of HFrEF patients do not achieve target dose of beta-blockade; greater use of strategies to reduce heart rate may improve outcomes in HFrEF.

## Background

Despite significant advances in care, HFrEF continues to have high rates of mortality and morbidity [1, 2]. Studies have shown that high resting heart rate is an independent risk factor for all-cause mortality, cardiovascular mortality, and cardiovascular events in the general population [3, 4], as well as in those with cardiovascular disease [5–7], coronary artery disease [8–10], hypertension [7, 11], heart failure [12], and diabetes [13]. The relation of heart rate to adverse outcomes may be mediated via its effects on coronary blood flow, cardiac contractility, and energy expenditure [5, 14]. These findings suggest that heart rate reduction may be an important target for clinicians to improve disease outcomes. A randomized controlled trial conducted in European patients with HFrEF, the Ivabradine and outcomes in Chronic Heart Failure (SHIFT) study, demonstrated that reduction of heart rate utilizing ivabradine is beneficial in HFrEF with heart rates > 70 bpm in spite of guideline directed therapy including beta blockers [15]. However, the relation of heart rate to outcomes in a real-world situation has not been well evaluated.

We conducted an observational study with long-term follow-up using national electronic health record data to examine the hypothesis that pulse rate (as an equivalent measure of heart rate), measured both around the time of diagnosis and repeatedly over time, is associated with mortality and hospitalization outcomes in HFrEF patients. Our results suggest that there is a strong, positive linear association between baseline, as well as serially measured pulse rate and risk of mortality and hospitalization outcomes, especially at values above 70 bpm.

## Methods

### Cohort selection

We identified incident cases of HFrEF diagnosed between 1/1/2006 and 12/31/2012 in the national Veterans Affairs (VA) healthcare system. Cases were defined as having an International Classification of Disease ninth revision (ICD-9) code for the diagnosis of heart failure (428.xx) and a left ventricular ejection fraction (LVEF) ≤35%. The date of the first occurrence of LVEF ≤35% was defined as the index date. To ensure these were incident cases, any patients who had a heart failure diagnosis code 1 year prior until 30 days before the index date in VA or in Centers for Medicare & Medicaid Services (CMS) data [16] were excluded. We excluded patients who had an atrial fibrillation or atrial flutter diagnosis prior to the index date not only to ensure presence of sinus rhythm, but also to ensure that pulse rate was equivalent to heart rate. To ensure sinus rhythm, we also excluded those who ever had a heart transplant or had paced rhythm within 6 months prior to the index date.

### Exposure

Baseline pulse rate (within 10 days after index date, outpatient or last inpatient value) and serially recorded time-varying pulse-rate, updated at 6-month intervals following the index date, were the exposures studied. For serial values, the first pulse rate measurement was recorded within the 6-month period after the index date with outcomes assessed from 6 through 12 months. Successive pulse values fell within the 6 months before the start of each subsequent 6-month window during which outcomes were assessed. Individual pulse rates < 30 or > 180 bpm were excluded. Pulse rates were considered equivalent to heart rate since they are generally verified by a physician or physician extender and corrected if there is a discrepancy between pulse rate measured by clinic staff and heart rate measured by clinical provider.

### Outcomes

The outcomes analyzed were all-cause mortality, and number of days hospitalized per year for any cause or for heart failure. Patients were followed in the VA databases supplemented by CMS data [16] from 6 months post index date for the longitudinal analyses and from the time of baseline pulse measurement for the baseline analyses. For both analyses, follow-up ended at death, last VA visit, or 12/31/2013. For the longitudinal analyses, outcomes were assessed within each 6-month interval following the 6-month interval in which pulse rate was measured. Patients with any inpatient stay longer than 180 days were excluded from the hospitalization outcome analyses.

### Covariates

Demographic, anthropometric, comorbidity, and laboratory variables were extracted from the VA healthcare system database. For the longitudinal analyses, time-varying covariates were updated at each 6-month interval with values that fell in the prior interval, closest to the start of the current interval. For the baseline analyses, covariates were selected within 30 days before and closest to the date of the baseline pulse rate measurement.

Prescription information for medication use was obtained from the pharmacy records within the VA healthcare system database and CMS [16]. We considered someone a user if they had a prescription dispensed within 6 months prior to the date of baseline pulse rate or the interval start date. We also categorized beta blocker use into < 50% target dose and ≥ 50% target dose, based on the type of beta blocker and dose at 1 year after diagnosis. Target doses were calculated for carvedilol, metoprolol succinate, and bisoprolol according to established treatment guidelines [17]; target doses were not analyzed for other beta blockers. Additional information regarding cohort selection, exposure, and covariates can be found in the supplementary methods.

### Statistical analysis

To evaluate pulse rate as a continuous predictor of all-cause mortality and hospitalization outcomes, we fit Cox proportional hazards and Poisson or negative binomial regression cubic spline plots, respectively. Generalized estimating equation (GEE) based negative binomial regression models with an ar (1) correlation structure were fit for hospitalization outcomes with longitudinal exposures, while Poisson models were fit for the baseline exposures. We used SAS macros to create natural cubic spline plots, specifying 5–7 knots equally distributed across the data, 3 degrees of freedom, and a reference value of 75 bpm.

Based on the relationship observed in the cubic spline plots, we categorized both repeated and baseline pulse rates by deciles: 47–59 bpm, 60–63 bpm, 64–67 bpm, 68–71 bpm, 72–75 bpm, 76–79 bpm, 80–83 bpm, 84–88 bpm, 89–96 bpm, and 97–117 bpm. Pulse rates that fell below the 1st percentile (47 bpm) and above the 99th percentile (117 bpm) were excluded. We selected 72–75 bpm as the reference group since the median and mean pulse values fell within that range and it is considered clinically normal. Additionally, we categorized elevated (≥70 bpm) and low pulse values (< 70 bpm). These decile and dichotomous categories were used for all proceeding regression analyses.

Regression models were built, starting with crude, then age-adjusted, parsimonious (age, gender, race), and finally multivariable models adjusted for age, gender, race, body mass index (BMI), estimated glomerular filtration rate (eGFR), serum potassium, serum sodium, use of angiotensin receptor blockers (ARBs), angiotensin converting enzyme inhibitors (ACEIs), nitrates, statins, aldosterone antagonists, calcium channel blockers, loop diuretics, and beta blockers, and history of coronary artery disease, hypertension, hyperlipidemia, chronic obstructive pulmonary disease, dementia, type II diabetes, stroke/ transient ischemic attack (TIA), cardiovascular disease, and anemia. Baseline multivariable models also adjusted for baseline LVEF and longitudinal analyses adjusted for development of atrial fibrillation or atrial flutter.

For both baseline and longitudinal analyses, Cox proportional hazards regression models were fit to examine the association between pulse rate category and all-cause mortality. Using a counting process approach [18], subjects entered the model at the time of their baseline pulse rate or the beginning of the 6-month interval, and person-time accrued until the end of the interval (for longitudinal analysis), death, last VA visit, or the end of follow-up (12/31/2013). Through examination of hazard and log negative log plots we concluded that the proportional hazards assumption was met. Hazard ratios (HR) and 95% confidence intervals (CI) were estimated for all pulse categories relative to the reference group.

GEE-based negative binomial regression models with an ar (1) correlation structure were fit to examine the association between longitudinal pulse rate categories and number of all-cause and heart failure hospitalization days per year. Person-observation-years were counted as the time (in years) that each subject was observed within each 6-month interval. Each subject could contribute to multiple pulse rate categories as their pulse values changed across 6-month intervals. The number of person-observation-years (on the log scale) was included as an offset in the models. Poisson regression models were fit to examine the association between baseline pulse rate and number of all-cause and heart failure hospitalization days per year, with years observed (on the log scale) included as an offset in the models. Rate ratios (RR) and 95% confidence intervals were estimated for all pulse rate categories relative to the reference group.

Our post-hoc sub-analysis included mortality cumulative incidence plots derived from multivariable adjusted Cox proportional hazards models stratified by pulse rate category to examine how survival varied by the interaction of beta blocker target dose ≥ and < 50% and pulse rate ≥ and < 70 bpm.

All analyses were conducted using SAS Enterprise Guide 7.1 (SAS institute Inc., Cary, NC). P–values are 2-tailed and significant at the alpha = 0.05 level.

## Results

**Fig. 1:**
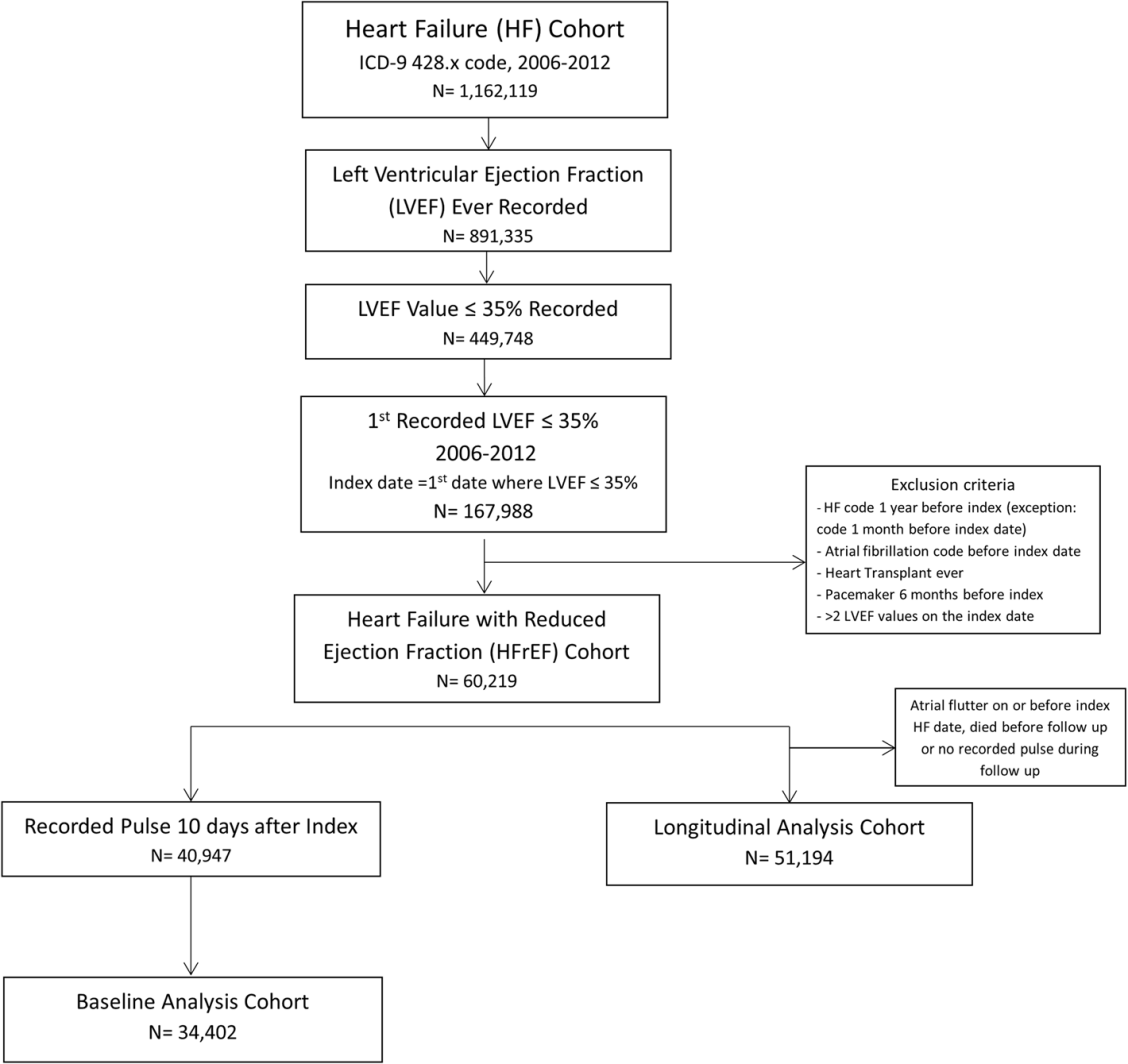
Flow chart for creation of Heart Failure with Reduced Ejection Fraction (HFrEF) cohorts

As shown in Fig. 1, the longitudinal analysis cohort (main cohort) and baseline analysis cohorts (subset that had baseline pulse rate values) consisted of 51,194 and 34,402 patients respectively. In the longitudinal analysis cohort, mean age was 67 ± 12 years, 98% were male, and 77% were white, as expected in a veteran population (Table 1). At the first 6-month time point, those in the lowest decile (47–59 bpm) were older, less likely to be African American, had lower eGFR, lower prevalence of COPD and diabetes, and higher prevalence of CAD and hyperlipidemia than those in the highest decile group (97–117 bpm). Similar patterns were seen across deciles for laboratory measurements and comorbidities at the 1.5-year timepoint (data not shown).

**Table 1:** Patient characteristics in first six-month interval after HFrEF diagnosis

Characteristics	Overall	1st Pulse Rate Decile	5th Pulse Rate Decile	10th Pulse Rate Decile	*p*-value
	*N* = 51,194	(47–59 bpm)	(72–75 bpm)	(97–117 bpm)	
		*n* = 7026	*n* = 5479	*n* = 2929	
Age, y	66.9 ± 11.5	69.4 ± 11	11.4 ± 62.5	62.5 ± 10.9	<.0001
Sex (male), %	50,267 (98.2%)	6949 (98.9%)	5378 (98.2%)	2841 (97.0%)	<.0001
Race,%					
White	35,577 (76.9%)	5061 (79.7%)	3782 (76.4%)	1833 (68.5%)	<.0001
African American	9784 (21.1%)	1170 (18.4%)	1062 (21.5%)	798 (29.8%)	
Other	912 (2.0%)	122 (1.9%)	106 (2.1%)	46 (1.7%)	
BMI,%					
< 18.5, kg/m^2^	1225 (2.4%)	131 (1.9%)	123 (2.3%)	137 (4.8%)	<.0001
18.5–24.9, kg/m^2^	12,766 (25.7%)	1959 (28.6%)	1360 (25.5%)	807 (28.2%)	
25–29.9, kg/m^2^	16,551 (33.3%)	2413 (35.2%)	1717 (32.2%)	816 (28.5%)	
30–34.9, kg/m^2^	10,998 (22.1%)	1425 (20.8%)	1221 (22.9%)	580 (20.3%)	
≥ 35, kg/m^2^	8203 (16.5%)	929 (13.6%)	915 (17.2%)	520 (18.2%)	
Baseline EF, %	26.6 ± 7.4	26.9 ± 7.4	26.6 ± 7.3	25.8 ± 7.5	<.0001
Serum Potassium, mEq/L	4.3 ± 0.5	4.4 ± 0.5	4.3 ± 0.5	4.3 ± 0.5	<.0001
Serum Sodium, mmol/L	138.9 ± 3.2	139.2 ± 2.9	138.9 ± 3.1	138.2 ± 3.3	<.0001
eGFR, mL/min/1.73 m^2^	67.7 ± 24.1	63.7 ± 22.9	67.5 ± 24	74.9 ± 25.6	<.0001
Anemia, %	24,707 (48.3%)	3591 (51.1%)	2693 (49.2%)	1591 (54.3%)	0.0001
Coronary artery disease, %	37,147 (72.6%)	5484 (78.1%)	4097 (74.8%)	1820 (62.1%)	<.0001
Chronic obstructive pulmonary disease, %	17,902 (35.0%)	2286 (32.5%)	1926 (35.2%)	1409 (48.1%)	<.0001
Dementia, %	5944 (11.6%)	865 (12.3%)	625 (11.4%)	493 (16.8%)	<.0001
Diabetes, %	25,011 (48.9%)	3155 (44.9%)	2839 (51.8%)	1596 (54.5%)	<.0001
Hypertension, %	42,866 (83.7%)	6155 (87.6%)	4671 (85.3%)	2483 (84.8%)	<.0001
Peripheral vascular disease, %	13,091 (25.6%)	1981 (28.2%)	1423 (26.0%)	760 (26.0%)	0.0025
Stroke/Transient ischemic attack, %	9494 (18.6%)	1547 (22.0%)	1025 (18.7%)	512 (17.5%)	<.0001
Hyperlipidemia, %	38,370 (75.0%)	5577 (79.4%)	4229 (77.2%)	2006 (68.5%)	<.0001
Atrial fibrillation/flutter, %	2673 (5.2%)	464 (6.6%)	301 (5.5%)	188 (6.4%)	<.0001

As shown in Table 2, within the first 6 months after the index date, 81% were on a beta blocker, 77% on an ACEI or ARB, and 16% on aldosterone antagonists. However, by 1.5 years the rates of use dropped to 73% for beta blocker, 67% for ACEI or ARB, and 15% for aldosterone antagonists. During the first 6 months after diagnosis, compared to the highest decile, patients in the lowest decile had higher use of beta blockers, statins, and nitrates, and lower use of loop diuretics. Use of ACEI or ARB varied less across deciles. Similar trends were seen across pulse rate deciles at 1.5 years for beta blocker, statin, and nitrate use. For many medications, the greatest decline in use between intervals was in the highest decile. No patients in our study were treated with ivabradine over the course of follow-up. Only 12.3% of patients received implantable cardioverter defibrillators (ICD) and only 4.2% underwent cardiac resynchronization therapy (CRT) following HFrEF diagnosis prior to the end of study follow-up.

**Table 2:** Medication Use in Intervals 1 (6 months after diagnosis) and 3 (1–1.5 years after diagnosis)

	Interval 1 (First 6 months)					Interval 3 (1–1.5 years)				
Characteristics	Overall	1st Pulse Rate Decile	5th Pulse Rate Decile	10th Pulse Rate Decile	*p*-value	Overall	1st Pulse Rate Decile	5th Pulse Rate Decile	10th Pulse Rate Decile	*p*-value
	*N* = 44,093	(47–59 bpm)	(72–75 bpm)	(97–117 bpm)		N = 44,093	(47–59 bpm)	(72–75 bpm)	(97–117 bpm)	
		*n* = 6116	*n* = 4753	*n* = 2269			*n* = 6433	*n* = 5094	*n* = 2214	
ACE Inhibitor or ARB, %	31,716 (77.2%)	4808 (78.6%)	3626 (76.3%)	1741 (76.7%)	0.0007	28,970 (67.0%)	4523 (70.3%)	3433 (67.4%)	1349 (60.9%)	<.0001
Aldosterone Antagonists, %	6574 (16.0%)	878 (14.4%)	789 (16.6%)	410 (18.1%)	<.0001	6374 (14.8%)	838 (13.0%)	778 (15.3%)	308 (13.9%)	<.0001
Beta blocker, %	33,407 (81.3%)	5177 (84.6%)	3861 (81.20%)	1696 (74.7%)	<.0001	31,735 (73.4%)	5031 (78.2%)	3804 (74.7%)	1373 (62.0%)	<.0001
Calcium channel blocker, %	8626 (21.0%)	1536 (25.1%)	945 (19.9%)	404 (17.8%)	<.0001	7861 (18.2%)	1442 (22.4%)	890 (17.5%)	353 (15.9%)	<.0001
Statins, %	29,996 (73.00%)	4644 (75.9%)	3488 (723.4%)	1480 (65.2%)	<.0001	28,950 (67.0%)	4579 (71.2%)	3401 (66.8%)	1253 (56.6%)	<.0001
Nitrates, %	8435 (20.5%)	1426 (23.3%)	1935 (19.7%)	397 (17.5%)	<.0001	7164 (16.6%)	1221 (19.0%)	830 (16.3%)	274 (12.4%)	<.0001
Loop Diuretic, %	20,508 (49.9%)	2847 (48.25%)	2394 (50.4%)	1287 (56.7%)	<.0001	18,575 (43.0%)	2642 (41.1%)	2243 (44.0%)	962 (43.5%)	0.0084
Thiazides, %	5838 (14.2%)	939 (15.4%)	666 (14.0%)	323 (14.2%)	0.1137	4670 (10.8%)	785 (12.2%)	540 (10.6%)	2055 (9.3%)	0.005
Anticoagulant, %	4017 (9.8%)	639 (10.4%)	456 (9.6%)	241 (10.6%)	0.1794	3715 (8.6%)	644 (10.0%)	407 (8.0%)	189 (8.5%)	<.0001
Digoxin, %	4410 (10.7%)	598 (9.8%)	518 (10.9%)	287 (12.6%)	0.0105	4176 (9.7%)	507 (7.9%)	511 (10.0%)	234 (10.6%)	<.0001

### Associations of baseline pulse rate with outcomes

**Fig. 2:**
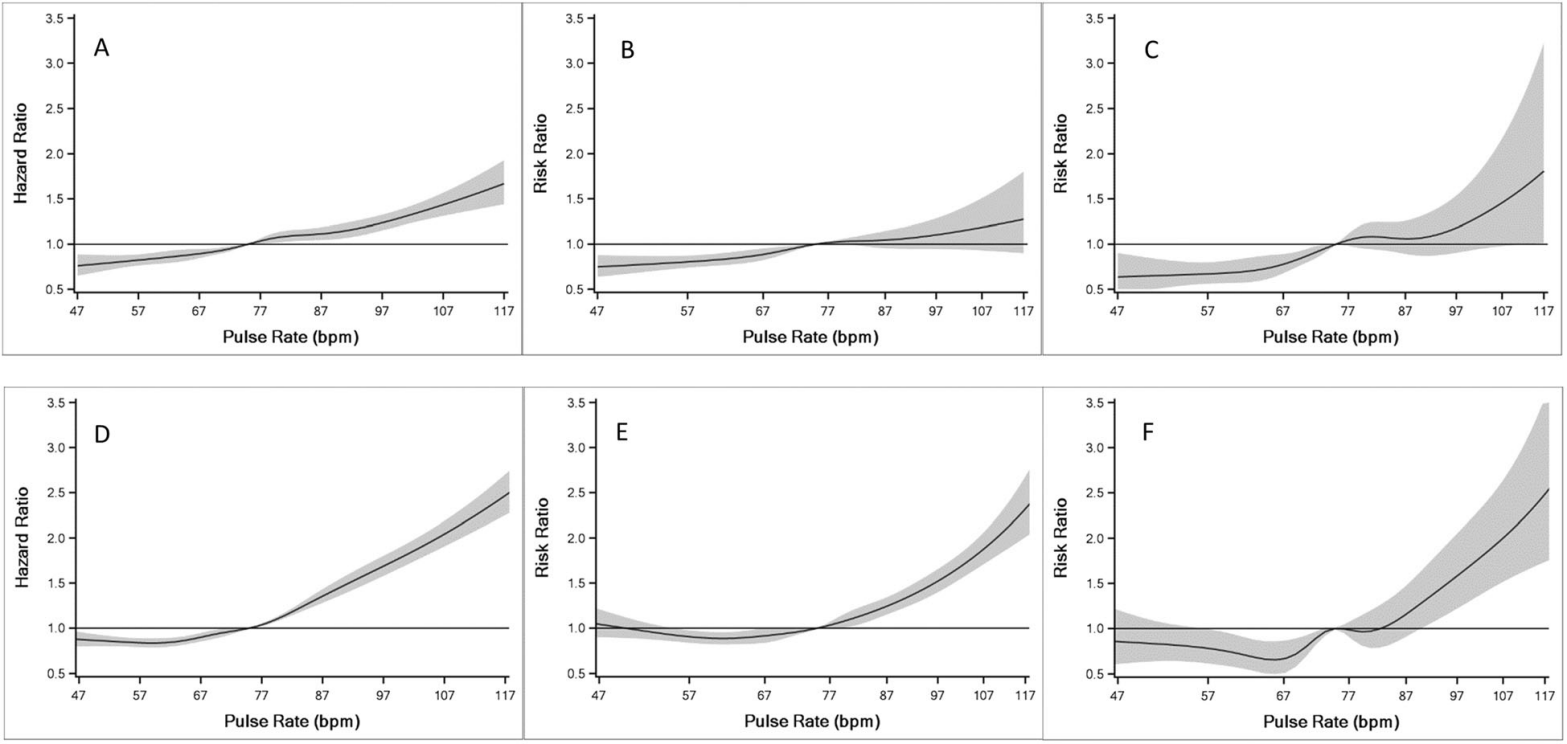
Cubic spline plots for multivariable adjusted association of outcomes with baseline and longitudinal pulse rates. (Central Illustration) Association of baseline pulse rate with (**a**) mortality using a Cox Proportional Hazards model, and (**b**) number of days all-cause hospitalizations per year, and (**c**) number of days heart failure hospitalizations per year using Poisson models. Associations of repeated pulse rates with (**d**) mortality using a Cox Proportional Hazards model, and (**e**) number of days all-cause hospitalizations per year, and (**f**) number of days heart failure hospitalizations per year using generalized estimating equation based negative binomial models. Bpm = beats per minute

As shown in Fig. 2a-c and Table 3, baseline pulse rate demonstrated significant positive, near linear associations with mortality and hospitalization outcomes. When comparing patients with baseline pulse rate ≥ 70 versus < 70, the following hazard and rate ratios were obtained: all-cause mortality - 1.26 (95% CI: 1.20–1.33); all-cause hospitalizations - 1.20 (95% CI: 1.12–1.28); and heart failure hospitalizations - 1.50 (95% CI: 1.27–1.77). Associations were significant in more pulse rate deciles for all-cause mortality and all-cause hospitalizations compared to heart failure hospitalizations (an outcome with lower event rates).

**Table 3:** Effect of Baseline and Longitudinal Pulse Rates on Outcomes

	Pulse rate (bpm)	Baseline Crude HR or RR^a^ (95% CI)	Baseline Multivariable^c^ HR or RR^a^ (95% CI)	Longitudinal Crude HR or RR^b^ (95% CI)	Longitudinal Multivariable^c^ HR or RR^b^ (95% CI)
**All-Cause Mortality (HR)**	47–59	0.91 (0.84–0.98) ^§^	0.85 (0.76–0.94) ^§^	0.92 (0.86–0.98) ^§^	0.84 (0.78–0.90) ^||^
	60–63	0.95 (0.88–1.02)	0.90 (0.81–1.00)	0.93 (0.87–0.99) ^§^	0.86 (0.80–0.94) ^§^
	64–67	0.99 (0.92–1.07)	0.92 (0.83–1.02)	0.92 (0.86–0.98) ^§^	0.88 (0.81–0.95) ^§^
	68–71	0.99 (0.92–1.07)	1.01 (0.92–1.12)	0.96 (0.90–1.02)	0.90 (0.83–0.97) ^§^
	72–75	–	–	–	–
	76–79	1.10 (1.02–1.18) ^§^	1.11 (1.01–1.22) ^§^	1.07 (0.99–1.14)	1.09 (1.00–1.18)
	80–83	1.07 (1.00–1.16)	1.14 (1.04–1.27) ^§^	1.10 (1.02–1.18) ^§^	1.11 (1.02–1.21) ^§^
	84–88	1.06 (0.99–1.15)	1.15 (1.04–1.27) ^§^	1.19 (1.11–1.28) ^||^	1.22 (1.12–1.33) ^||^
	89–96	1.13 (1.05–1.22) ^§^	1.25 (1.14–1.38) ^||^	1.41 (1.32–1.52) ^||^	1.55 (1.42–1.68) ^||^
	97–117	1.21 (1.12–1.30) ^||^	1.43 (1.30–1.58) ^||^	1.84 (1.71–1.97) ^||^	2.03 (1.86–2.21) ^||^
**All-Cause Hospitalizations (RR)**	47–59	0.79 (0.70–0.90) ^§^	0.84 (0.73–0.97) ^§^	0.95 (0.87–1.02)	1.00 (0.90–1.10)
	60–63	0.91 (0.80–1.03)	0.98 (0.85–1.13)	0.91 (0.84–0.99) ^§^	0.93 (0.85–1.03)
	64–67	0.92 (0.81–1.04)	0.96 (0.84–1.10)	0.90 (0.83–0.98) ^§^	0.89 (0.81–0.98) ^§^
	68–71	0.94 (0.84–1.06)	1.02 (0.90–1.16)	0.96 (0.89–1.04)	1.02 (0.92–1.13)
	72–75	–	–	–	–
	76–79	1.12 (0.99–1.26)	1.16 (1.02–1.31) ^§^	1.01 (0.93–1.09)	1.06 (0.95–1.18)
	80–83	1.21 (1.08–1.36) ^||^	1.19 (1.04–1.35) ^§^	1.17 (1.07–1.28) ^§^	1.22 (1.09–1.37) ^§^
	84–88	1.24 (1.10–1.39) ^||^	1.18 (1.04–1.34) ^§^	1.18 (1.09–1.29) ^||^	1.19 (1.06–1.34) ^§^
	89–96	1.28 (1.14–1.44) ^||^	1.11 (0.98–1.26)	1.44 (1.32–1.56) ^||^	1.43 (1.29–1.59) ^||^
	97–117	1.48 (1.32–1.65) ^||^	1.30 (1.14–1.47) ^||^	2.06 (1.89–2.25) ^||^	1.85 (1.66–2.07) ^||^
**Heart Failure Hospitalizations (RR)**	47–59	0.68 (0.49–0.93) ^§^	0.68 (0.48–0.97) ^§^	0.79 (0.57–1.08)	0.88 (0.66–1.17)
	60–63	0.77 (0.56–1.06)	0.87 (0.62–1.23)	0.84 (0.59–1.19)	0.97 (0.70–1.36)
	64–67	0.72 (0.52–0.99) ^§^	0.73 (0.52–1.03)	0.65 (0.47–0.89) ^§^	0.66 (0.50–0.86) ^§^
	68–71	0.96 (0.73–1.28)	1.02 (0.76–1.38)	0.86 (0.59–1.26)	0.98 (0.72–1.33)
	72–75	–	–	–	–
	76–79	1.18 (0.89–1.56)	1.23 (0.91–1.64)	0.92 (0.65–1.30)	1.19 (0.81–1.76)
	80–83	1.27 (0.97–1.68)	1.20 (0.89–1.62)	1.05 (0.75–1.45)	1.20 (0.90–1.61)
	84–88	1.38 (1.06–1.80) ^§^	1.23 (0.92–1.64)	1.06 (0.76–1.46)	1.24 (0.92–1.68)
	89–96	1.33 (1.01–1.74) ^§^	1.09 (0.81–1.46)	1.38 (1.00–1.92)	1.61 (1.18–2.21) ^§^
	97–117	1.85 (1.43–2.39) ^||^	1.64 (1.24–2.17) ^§^	2.11 (1.53–2.92) ^||^	2.29 (1.65–3.17) ^||^

*HR* Hazard Ratio, *RR* Rate Ratio, *bpm* beats per minute

^a^Poisson regression model

^b^Negative Binomial regression model

^**c**^Multivariable models were adjusted for age, gender, race, BMI, eGFR, serum potassium, serum sodium, use of beta blockade, ARBs, ACEIs, nitrates, statins, aldosterone antagonists, calcium channel blockers, loop diuretics, and history of coronary artery disease, hypertension, hyperlipidemia, chronic obstructive pulmonary disease, dementia, diabetes, stroke/TIA, cardiovascular disease and anemia. The baseline multivariate model was additionally adjusted for baseline LVEF and the longitudinal multivariate was adjusted for development of atrial fibrillation or atrial flutter

§*p* < .05 ||*p* < .0001

### Association of longitudinal pulse rate with all-cause mortality

Over a median follow-up of 3.2 years (IQR: 1.6–5.1) in the longitudinal analysis cohort, 16,370 deaths occurred; during the first 6 months after heart failure diagnosis, an additional 3844 patients died who were not included in the analysis, as follow-up started at 6 months.

As shown in Fig. 2d, repeated pulse rate measures demonstrated a positive linear relationship with all-cause mortality above a pulse rate of around 70 bpm. When comparing patients who had a pulse rate ≥ 70 in the prior 6-month interval versus those with a pulse rate < 70, we observed a hazard ratio of 1.36 (95% CI: 1.31–1.42) for all-cause mortality. Table 3 presents the association of repeated pulse rate measurements categorized into deciles with all outcomes.

### Associations of longitudinal pulse rate with all-cause hospitalizations

As shown in Fig. 2e and Table 3, a significant, positive linear association was observed between repeated pulse rate measurements ≥80 bpm and number of days all-cause hospitalization per year. Patients who had a pulse rate ≥ 70 bpm in the past 6 months had 1.25 times (95% CI: 1.19–1.32) increased rate of hospitalizations compared to those with pulse rate < 70 bpm.

### Associations of longitudinal pulse rate with heart failure hospitalizations

A positive, linear relationship was demonstrated for repeated pulse rate measurements ≥80 bpm and number of days hospitalized for heart failure per year (Fig. 2f and Table 3), with statistically significant increases noted in the 9th and 10th deciles. Patients who had a pulse rate ≥ 70 bpm in the past 6 months had 1.51 times (95% CI: 1.33–1.72) increased rate of hospitalizations for heart failure per year compared to those with pulse rate < 70 bpm.

### Effect of Beta blocker dose

**Fig. 3:**
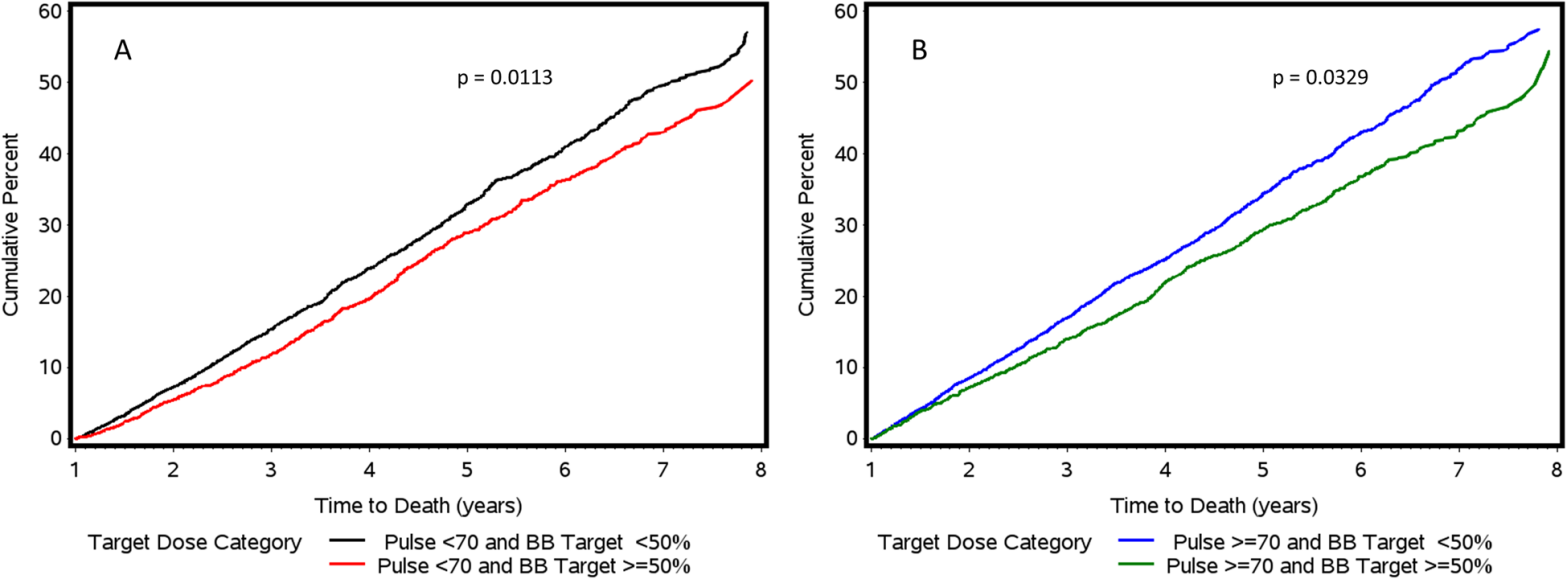
Cumulative mortality by beta blocker target dose for (**a**) pulse < 70, (**b**) ≥70 bpm. Beta-blocker target dose was assessed 1 year after HFrEF diagnosis and analyses are based on multivariable adjusted Cox proportional hazards models. bb = beta blocker; HFrEF=Heart Failure with Reduced Ejection Fraction; bpm = beats per minute

Only 19,453 patients were on guideline recommended beta blockers, of whom only 7915 (15% of total cohort) were on ≥50% target doses. Figure 3 demonstrates the cumulative incidence of all-cause mortality by beta blocker dose (≥or < 50% target dose) for patients with pulse rate < 70 and ≥ 70 bpm at 1 year after the index date. Among patients with pulse rate < 70 bpm, those who achieved ≥50% beta blocker target dose had significantly improved mortality outcomes than those at < 50% beta blocker target dose (*p* = 0.0113). Similarly, among patients with pulse rate ≥ 70 bpm, those who achieved ≥50% beta blocker target dose had significantly better survival than those at < 50% beta blocker target dose (*p* = 0.0329).

## Discussion

Our study, which examined the associations of pulse rate measured from the time of index diagnosis of HFrEF in a large, national cohort, demonstrated a significant association of both baseline pulse rate and pulse rate measured repeatedly over follow-up with morbidity and mortality in heart failure. Our study also demonstrated that guideline directed beta-blocker use was sub-optimal in a real world setting and that higher heart rate was associated with adverse outcomes independent of beta blocker use.

Similar to previous studies [19–23], we found that patients in higher pulse categories were younger, had lower baseline LVEF values, lower beta blocker use, and higher use of loop diuretics and aldosterone antagonists. The prognostic value of a single heart rate measurement at the time of heart failure diagnosis is not well-known; in our study, baseline pulse rate was positively associated with mortality and hospitalizations. A possible reason is that a high heart rate may be associated with greater neurohormonal activation and thus worse outcomes independent of subsequent treatment. It is also possible that this association is the result of a lack of intensification of therapy with neurohormonal antagonists in the real-world setting, since the proportion of patients receiving therapy did not increase significantly during follow-up, similar to recent reports [24]. Sub-analyses from the Candesartan in Heart Failure-Assessment of Reduction in Mortality and Morbidity (CHARM) trial, placebo arm of the SHIFT trial, and pooled data from the SHIFT and Ivabradine for patients with coronary artery disease and left-ventricular systolic dysfunction (BEAUTIFUL) trials also showed that as baseline heart rate (albeit at time of entry into clinical trial and not at index diagnosis of HFrEF) increased, there was a significant increased risk of all-cause mortality, as well as the composite outcome of cardiovascular death or HF hospitalization [15, 19, 25]. Similarly, a meta-analysis of 11 randomized controlled trials of beta blockers in HF patients in sinus rhythm showed a significant positive linear association between heart rate at time of enrollment and all-cause mortality [22]. Also in accordance with our findings, Ibrahim et al., who examined associations of heart rate in a single center cohort from baseline (first visit where patients had LVEF≤35% and sinus rhythm), demonstrated a positive association between baseline heart rate and all-cause mortality or HF hospitalization [21]. However, a post-hoc analysis from the Efficacy of Vasopressin Antagonism in Heart Failure Outcome Study with Tolvaptan (EVEREST) trial showed that baseline heart rate was not significantly associated with all-cause mortality (*p* ≥ 0.066) in HFrEF patients in sinus rhythm [20]. The results of our study in a large U.S. real-world population, conclusively demonstrate the predictive value of pulse rate measured at the time of diagnosis of HFrEF. In the meta-analysis of clinical trials described above, heart rates at interim visits (mean 184 days after randomization and unknown time relative to diagnosis) were more predictive of mortality than change in heart rate from randomization [22]. Greene et al., in their post-hoc analysis of the EVEREST trial mentioned above [20], found that heart rates ≥70 bpm post-discharge (only through 4 weeks) were associated with increased risk of mortality. Similar to our serial measurement approach, Hamill and colleagues studied the effect of heart rate at time of entry into clinical trial and of repeated heart rate measurements, albeit only in the year following enrollment [25]. Their cohort only included 8,699 patients, but still found a strong association between baseline heart rate and HF hospitalization and cardiovascular death, with associations strengthened for repeated heart rate measurements. Our study was much larger, including only HFrEF patients, and had a longer median follow-up period of 3.2 years from a true baseline timepoint.

Most studies that have looked at the association between heart rate and outcomes were post-hoc analyses of clinical trials. Patients were only eligible to enroll in many trials if they had a heart rate ≥ 60 bpm or ≥ 70 bpm [23, 25, 26], while in our real-world study we examined patients with pulse rates as low as 47 bpm. In previous studies, repeated heart rate measurements were obtained only during study mandated visits often limited to one follow-up visit within the first year after study enrollment [20, 22, 25], while we had an average of 6.3 ± 3.6 pulse measurements per patient updated at 6 month intervals over a median follow-up of 3.2 years. Previous studies except one single-center observational cohort study [21] also defined baseline as the time of randomization and enrollment into the clinical trial, which is unlikely to have been a true “baseline” at the time of index diagnosis.

Although multiple studies have shown that heart rate reduction is associated with improved clinical outcomes in heart failure patients [26–31], it remains controversial whether benefits from beta blockers are stronger due to achievement of target doses that help to reduce heart rate [32] or from the magnitude of heart rate reduction [27–29, 33]. In our study, only 15% achieved ≥50% target dose of guideline directed beta blockade. Similar to other studies [21, 22], our study also demonstrated that a lower pulse rate is associated with better outcomes in HFrEF independent of treatment with beta-blockade. However, in patients who were on guideline directed beta blockers, our results suggest that achieving at least 50% of target dose was also an important predictor of better outcomes at both low and high pulse rates. While both lower heart rate and higher beta blocker target dose are associated with outcomes in heart failure patients, further studies are necessary to better understand their roles together in improving outcomes. Although we were unable to assess the effect of ivabradine in our cohort, it warrants further investigation into how effectively it improves heart failure outcomes through reduction of heart rate in real world settings relative to or in combination with beta-blockers [15, 34].

### Strengths and limitations

There are several major strengths of this real-world study. This study was done in a large national cohort; baseline pulse rates close to the index diagnosis of HFrEF were used for analyses; serial pulse rates and three major outcomes were measured over a long period of follow-up; and electronic health records were mined to obtain detailed and serial clinical, laboratory, and medication data. Inclusion of a significant number of African Americans also lends strength to this study. However, there were some limitations. Since this was a retrospective cohort study, there remains potential for unmeasured confounding after model adjustment. To minimize the potential for bias in retrospective studies, the study population and exposure categories were systematically defined, and we captured outcomes after diagnosis using data from electronic medical records. There was potential for differential misclassification since patients who were sicker likely received more medical care and thus had more pulse measurements, however a single measurement was observed at baseline and all longitudinal pulse rates were updated at regular 6-month intervals allowing patients to oscillate between categories and carrying forward any value recorded in the prior year. As expected in a VA cohort, there was a high proportion of white males, however we adjust for both race and gender in our analyses. To account for care received outside the VA system, we utilized data from CMS; though it is possible that we may not have captured the entirety of care received outside the VA.

## Conclusions

A lower pulse rate at the time of HFrEF diagnosis and across follow-up clinical encounters is strongly associated with lower risk of mortality and hospitalization outcomes in a real-world setting, independent of the use of beta blockade. Strategies to lower heart rate, including use of beta blockade, may be beneficial in reducing the morbidity and mortality associated with HFrEF.

## Supplementary information

**Additional file 1.** Contains additional information regarding the methods used.

## Data Availability

The data that support the findings of this study are available from the United States Department of Veterans Affairs corporate data warehouse, but since this project was not federally funded and in accordance with current VA policies, restrictions apply to the availability of these data, which were used for the current study, and so are not publicly available. Methods used in analysis, however, may be provided to any researcher for purposes of replicating the procedure upon request from the senior author.

## Abbreviations

ACEI: Angiotensin converting enzyme inhibitors
ARB: Angiotensin receptor blockers
BEAUTIFUL: Ivabradine for patients with coronary artery disease and left-ventricular systolic dysfunction trial
BMI: Body mass index
BPM: Beats per minute
CHARM: Candesartan in heart failure-assessment of reduction in mortality and morbidity trial
CI: Confidence interval
CMS: Centers for Medicare & Medicaid
CPT: Current procedural terminology
eGFR: Estimated glomerular filtration rate
EVEREST: Efficacy of vasopressin antagonism in heart failure outcome study with Tolvaptan trial
GEE: Generalized estimating equation
HFrEF: Heart failure with reduced ejection fraction
HR: Hazard ratio
ICD-9: *International classification of disease ninth revision*
LVEF: Left ventricular ejection fraction
NLP: Natural language processing
RR: Rate ratio
SHIFT: Ivabradine and outcomes in chronic heart failure
TIA: Transient ischemic attack
TIU: Text integration utilities
VA: Veterans affairs
