# Supplementary material for: Association of pulse rate with outcomes in heart failure with reduced ejection fraction: a retrospective cohort study
Source: BMC Cardiovasc Disord. 2020 Feb 26;20:92. doi: 10.1186/s12872-020-01384-6 (PMC7045436; doi:10.1186/s12872-020-01384-6)
Supplement: Supplementary file 1 — Additional file 1. Contains additional information regarding the methods used. [file 12872_2020_1384_MOESM1_ESM.docx]

**Supplementary Methods**

*Ejection Fractions*

A Natural Language Processing (NLP) tool, developed and validated at the VA (1), was used to extract LVEF values from VA Text Integration Utilities (TIU) documents, which include history and physical examination notes, progress notes, discharge summary notes, echocardiography reports, nuclear medicine reports, cardiac catheterization reports, and other cardiology notes (1, 2). Patients who had multiple LVEF measurements recorded on their index date that were more than 5% apart were excluded from our analysis. Since it is uncommon to have more than one echocardiogram in the same day, these were likely NLP errors.

*Exclusion Criteria*

As it has previously been shown that there is no association between heart rate and adverse cardiovascular outcomes in patients with atrial flutter or atrial fibrillation (3, 4), we excluded patients with these conditions at baseline based on ICD-9 diagnosis codes (427.31, 427.32). For exclusions due to heart transplant, ICD-9 diagnosis code v42.1, ICD-9 procedure codes 33.6, 37.5x, 37.62, 37.63, 37.65, 37.66, 37.68, and CPT codes 0051T, 0052T, 0053T, 33930, 33933, 33935, 33940, 33944, 33945, 33975, 33976, 33977, 33978, 33979, 33990, 33992, 33993 were used. Finally, ICD-9 diagnosis code v45.01, ICD-9 procedure codes 00.50, 00.53, 37.71, 37.72, 37.73, 37.75, 37.76, 37.77, 37.79, 37.80, 37.81, 37.82, 37.83, 37.85, 37.86, 37.87, 37.89, 37.98, and CPT codes 33206, 33207, 33208, 33212, 33213, 33214, 33216, 33217, 33221, 33224, 33225, were used to identify and exclude patients who had a recent paced rhythm.

*Exposure*

When selecting baseline pulse rates, preference was given to outpatient values, since they’re likely to be more stable than inpatient values. If outpatient pulse rates were not available within 10 days of the index date, the last recorded pulse rate during an inpatient stay was selected. For those diagnosed during inpatient stays, if no pulse was measured on the discharge date or the discharge date was more than 10 days after the index date, the patient was excluded.

For time-varying pulse rates, the minimum outpatient pulse value recorded within 30 days prior to the start of the 6-month follow-up window was selected. If no outpatient pulse was recorded within this 30-day window, the outpatient or inpatient discharge value recorded before and closest to the interval start date was selected, with prioritization of outpatient values. If there were no pulse values recorded during the 6-month interval, then the last value recorded was carried forward from previous intervals.

For both analyses, if multiple pulse rates were recorded on the same day, the minimum outpatient or the last recorded inpatient value was used.

*Covariate Selection*

Covariates, including age, gender, race, body mass index (BMI), baseline LVEF, serum sodium, serum potassium, serum creatinine, and hemoglobin (used to assess anemia) were extracted from the VA healthcare system database. Races that were not white or black were reported as “other” due to low frequencies in the Asian, American Indian, and Pacific Islander standard race categories. BMI was categorized using the World Health Organization international classification (5). Estimated glomerular filtration rate (eGFR) was calculated from serum creatinine, age, and race using the chronic kidney disease epidemiology collaboration (CKD-EPI) formula (6).

Prescription information for angiotensin converting enzyme inhibitors (ACEI), angiotensin receptor blockers (ARB), beta blockers, nitrates, statins, calcium channel blockers, aldosterone antagonists, loop diuretics, thiazides, anticoagulants, and digoxin was obtained from the VA pharmacy records and CMS(7).

Comorbid conditions were also identified using ICD-9 diagnosis codes from both the VA and CMS databases(7). To qualify as having a condition, patients were required to have either one inpatient code or two outpatient codes prior to the interval start date or date of baseline pulse rate. The following ICD-9 diagnosis codes were used to identify comorbid conditions: 410-414, V45.82 for coronary artery disease including myocardial infarction, percutaneous coronary intervention, or coronary artery bypass graft; 401-405, 437.2 for hypertension; 272 hyperlipidemia; 490-496, 510, 781.5 for chronic obstructive pulmonary disease; 290-294, 330-331.2, 331.82, 797 for dementia; 250, 362.0, 357.2, 366.41, 249, 790.2-791.6, V45.85, V53.91, V65.46 for type II diabetes; 430-438 for stroke or transient ischemic attack; 390–394, 429–448 for cardiovascular disease; and 280-285 and/or low hemoglobin values (<13.5 for males and <12 for females) anemia. Incident atrial fibrillation or atrial flutter (427.31, 427.32) were also examined for the longitudinal analyses.

**References**

1. Patterson OV, Freiberg MS, Skanderson M, S JF, Brandt CA, DuVall SL. Unlocking echocardiogram measurements for heart disease research through natural language processing. BMC Cardiovasc Disord. 2017;17(1):151.

2. Patel YR, Robbins JM, Kurgansky KE, Imran T, Orkaby AR, McLean RR, et al. Development and validation of a heart failure with preserved ejection fraction cohort using electronic medical records. BMC Cardiovasc Disord. 2018;18(1):128.

3. Castagno D, Skali H, Takeuchi M, Swedberg K, Yusuf S, Granger CB, et al. Association of heart rate and outcomes in a broad spectrum of patients with chronic heart failure: results from the CHARM (Candesartan in Heart Failure: Assessment of Reduction in Mortality and morbidity) program. J Am Coll Cardiol. 2012;59(20):1785-95.

4. Kotecha D, Flather MD, Altman DG, Holmes J, Rosano G, Wikstrand J, et al. Heart Rate and Rhythm and the Benefit of Beta-Blockers in Patients With Heart Failure. J Am Coll Cardiol. 2017;69(24):2885-96.

5. WHO. Obesity: preventing and managing the global epidemic. Report of a WHO Consultation. WHO Technical Report Series 894. Geneva: World Health Organization; 2000.

6. Levey AS, Stevens LA, Schmid CH, Zhang Y, Castro AF, Feldman HI, et al. A New Equation to Estimate Glomerular Filtration Rate. Annals of Internal Medicine. 2009;150(9):604-12.

7. US Department of Veterans Affairs. System of Records Notice 97VA10P1: Consolidated Data Information System-VA. 76 FR 25409. Dec 31, 2013.
